# The 1:2 co-crystal formed between *N*,*N*′-bis(pyridin-4-ylmeth­yl)ethanedi­amide and benzoic acid: crystal structure, Hirshfeld surface analysis and computational study

**DOI:** 10.1107/S2056989019016840

**Published:** 2020-01-01

**Authors:** Sang Loon Tan, Edward R. T. Tiekink

**Affiliations:** a Research Centre for Crystalline Materials, School of Science and Technology, Sunway University, 47500 Bandar Sunway, Selangor Darul Ehsan, Malaysia

**Keywords:** crystal structure, oxalamide, hydrogen bonding, Hirshfeld surface analysis, computational chemistry

## Abstract

The crystal and mol­ecular structures of the title 1:2 co-crystal, C_14_H_14_N_4_O_2_·2C_7_H_6_O_2_, are described. The oxalamide mol­ecule has a (+)-anti­periplanar conformation with the 4-pyridyl residues lying to either side of the central, almost planar C_2_N_2_O_2_ chromophore (r.m.s. deviation = 0.0555 Å). The benzoic acid mol­ecules have equivalent, close to planar conformations [C_6_/CO_2_ dihedral angle = 6.33 (14) and 3.43 (10)°]. The formation of hy­droxy-O—H⋯N(pyrid­yl) hydrogen bonds between the benzoic acid mol­ecules and the pyridyl residues of the di­amide leads to a three-mol­ecule aggregate. Centrosymmetrically related aggregates assemble into a six-mol­ecule aggregate *via* amide-N—H⋯O(amide) hydrogen bonds through a 10-membered {⋯HNC_2_O}_2_ synthon. These are linked into a supra­molecular tape *via* amide-N—H⋯O(carbon­yl) hydrogen bonds and 22-membered {⋯HOCO⋯NC_4_NH}_2_ synthons. The contacts between tapes to consolidate the three-dimensional architecture are of the type methyl­ene-C—H⋯O(amide) and pyridyl-C—H⋯O(carbon­yl). These inter­actions are largely electrostatic in nature. Additional non-covalent contacts are identified from an analysis of the calculated Hirshfeld surfaces.

## Abstract 2

The 4-pyridyl residues lie to either side of the central, planar C_2_N_2_O_2_ chromophore of the oxalamide mol­ecule which has a + anti-periplanar conformation. Conventional hydrogen-bonding inter­actions lead to supra­molecular tapes in the crystal.

## Chemical context   

Co-crystal technology continues to attract significant attention in a variety of endeavours with quite likely the most important of these relating to the development of more efficacious drugs by non-covalent derivatization of active pharmaceutical ingredients (Duggirala *et al.*, 2016 ▸; Bolla & Nangia, 2016 ▸; Gunawardana & Aakeröy, 2018 ▸). In order to predictably form co-crystals, reliable and robust synthons are needed. One such synthon is that formed by a carb­oxy­lic acid and a pyridyl residue *via* an O—H⋯N hydrogen bond (Shattock *et al.*, 2008 ▸). Very high propensities were noted, *i.e*. in the mid- to high-90%, in instances where there were no competing supra­molecular synthons involving hydrogen bonding (Shattock *et al.*, 2008 ▸). This compares to 33% adoption of the more familiar eight-membered {⋯OCOH}_2_ homosynthon by carb­oxy­lic acids (Allen *et al.*, 1999 ▸). This high propensity for O—H⋯N hydrogen-bond formation pertains to bis­(pyridin-*n-*ylmeth­yl)ethanedi­amide mol­ecules, *i.e*. species with the general formula *n*-NC_5_H_4_CH_2_N(H)C(=O)C(=O)CH_2_C_5_H_4_N-*n*, for *n* = 2, 3 and 4, hereafter abbreviated as *^n^L*H_2_. Indeed, in early studies on crystal engineering, the combination of bifunctional *^n^L*H_2_ with di­carb­oxy­lic acids such as bis­(carb­oxy­meth­yl)oxalamide (Nguyen *et al.*, 1998 ▸) and bis­(carb­oxy­meth­yl)urea (Nguyen *et al.*, 2001 ▸) enabled the systematic construction of two-dimensional arrays. As part of a long-term inter­est in the structural chemistry of *^n^L*H_2_ (Tiekink, 2017 ▸) and of systematic investigations of acid–pyridine co-crystals (Arman, Kaulgud *et al.*, 2012 ▸; Arman & Tiekink, 2013 ▸; Arman *et al.*, 2013 ▸, 2014 ▸), the title 1:2 co-crystal formed between ^4^ *L*H_2_ and benzoic acid was characterized by X-ray crystallography and the supra­molecular association further probed by Hirshfeld surface analysis and computational chemistry.
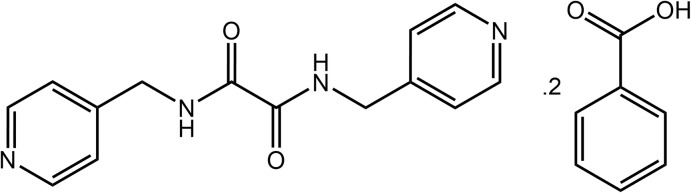


## Structural commentary   

**Figure 1:**
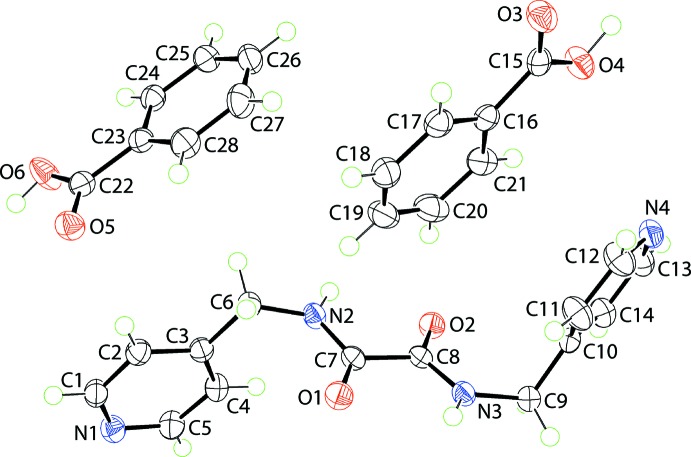
The mol­ecular structures of the constituents of (I) showing the atom-labelling scheme and displacement ellipsoids at the 70% probability level.

The mol­ecular structures of the three constituents comprising the crystallographic asymmetric unit of (I) are shown in Fig. 1 ▸. The ^4^ *L*H_2_ mol­ecule lacks crystallographic symmetry but adopts a (+)-antiperiplanar conformation where the 4-pyridyl residues lie to either side of the central C_2_N_2_O_2_ chromophore. The six atoms comprising the central residue are close to co-planar with their r.m.s. deviation equal to 0.0555 Å, with the maximum deviations to either side of the plane being 0.0719 (5) and 0.0642 (5) Å for the N2 and O2 atoms, respectively; the C6 and C9 atoms lie 0.1908 (14) and 0.0621 (14) Å out of and to one side of the plane (towards the N2 atom), respectively. The N1- and N4-pyridyl rings form dihedral angles of 86.00 (3) and 83.34 (2)°, respectively, with the plane through the C_2_N_2_O_2_ atoms, so are close to perpendicular to the central plane. The dihedral angle between the pyridyl rings is 33.60 (5)°, indicating a splayed disposition as each pyridyl ring is folded away from the rest of the mol­ecule. The carbonyl groups are *anti* and the mol­ecule features intra­molecular amide-N—H⋯O(carbon­yl) hydrogen bonds that complete *S*(5) loops, Table 1 ▸.

There are two independent benzoic acid mol­ecules in (I). Each is approximately planar with the dihedral angle between the benzene ring and CO_2_ group being 6.33 (14) and 3.43 (10)° for the O3- and O5-benzoic acid mol­ecules, respectively. As expected, the C15—O3(carbon­yl) bond length of 1.2162 (13) Å is significantly shorter than the C15—O4(hy­droxy) bond of 1.3197 (13) Å; the bonds of the O5-benzoic acid follow the same trend with C22—O5 of 1.2237 (13) Å compared with C22—O6 of 1.3084 (13) Å.

## Supra­molecular features   

**Figure 2:**
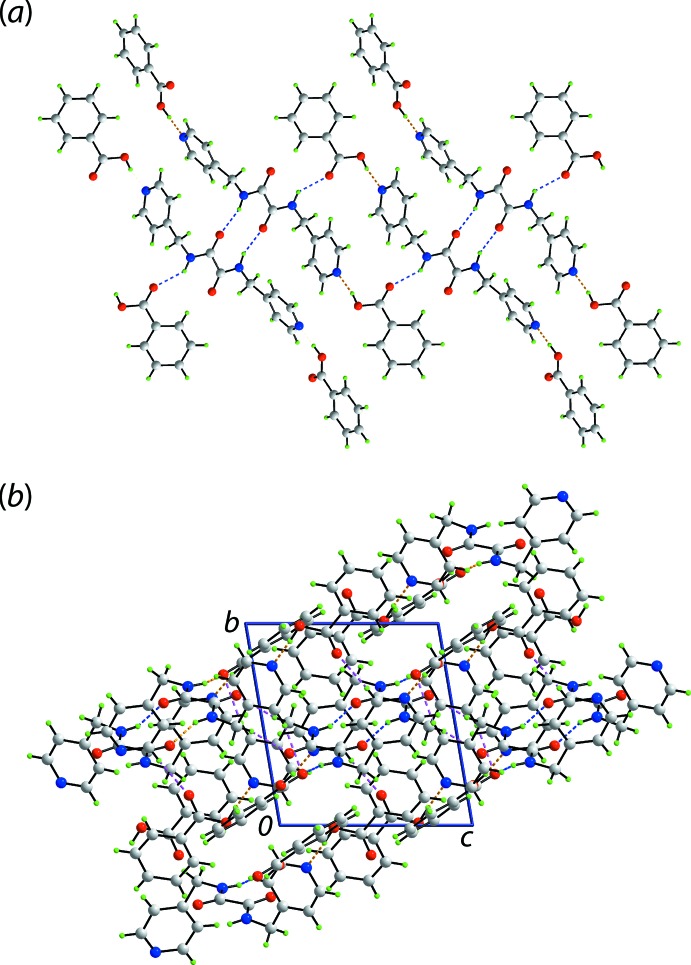
Mol­ecular packing in the crystal of (I): (*a*) supra­molecular tape sustained by hy­droxy-O—H⋯N(pyrid­yl) (orange dashed lines) and amide-N—H⋯O(amide, carbon­yl) hydrogen bonds and (*b*) a view of the unit-cell contents in projection down the *c* axis with C—H⋯O inter­actions shown as pink dashed lines.

As anti­cipated from the chemical composition, significant conventional hydrogen bonding is noted in the crystal of (I) over and above the intra­molecular amide-N—H⋯O(carbon­yl) hydrogen bonds already noted. The geometric parameters characterizing the specified inter­molecular contacts are listed in Table 1 ▸. The most prominent feature in the crystal is the formation of the expected three-mol­ecule aggregate sustained by hy­droxy-O—H⋯N(pyrid­yl) hydrogen bonding. This is connected into a six-mol­ecule aggregate *via* amide-N—H⋯O(amide) hydrogen bonding, which leads to a centrosymmetric ten-membered {⋯HNC_2_O}_2_ synthon. The second amide forms an amide-N—H⋯O(carbon­yl) bond with the result of that adjacent six-mol­ecule aggregates are connected into a supra­molecular tape *via* 22-membered {⋯HOCO⋯NC_4_NH}_2_ synthons, Fig. 2 ▸(*a*). The other notable contact within the tape is a pyridyl-C—H⋯O(carbon­yl) inter­action, which cooperates with a hy­droxy-O—H⋯N(pyrid­yl) hydrogen bond to form a seven-membered {⋯OCOH⋯NCH} pseudo-heterosynthon; no analogous inter­action is noted for the O5-benzoic acid. The supra­molecular tapes are aligned along the *c*-axis direction and have a linear topology.

The connections between chains leading to a three-dimensional architecture are of the type C—H⋯O, *i.e*. methyl­ene-C—H⋯O(amide) and pyridyl-C—H⋯O(carbon­yl), the latter involving both pyridyl rings and each carbonyl-O atom, Table 1 ▸ and Fig. 2 ▸(*b*).

## Hirshfeld surface analysis   

**Figure 3:**
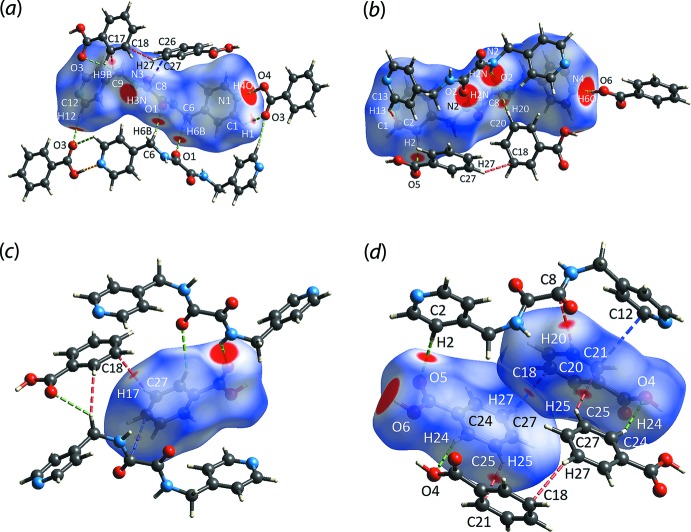
The *d*_norm_ map showing N—H⋯O (yellow dashed line), C—H⋯O (green), C—H⋯C (red) and C⋯C (blue) close contacts as indicated by the corresponding red spots with varying intensities within the range of −0.1004 to 1.1803 arbitrary units for (*a*) ^4^ *L*H_2_, (*b*) ^4^ *L*H_2_ viewed from a different perspective, (*c*) BA-II and (*d*) BA-II (left) and BA-I (right).

The program *Crystal Explorer 17* (Turner *et al.*, 2017 ▸) was used for the calculation of the Hirshfeld surfaces and two-dimensional fingerprint plots based on the procedures described previously (Tan, Jotani *et al.*, 2019 ▸). The three-mol­ecule aggregate whereby the two benzoic acid (BA) mol­ecules are connected to ^4^ *L*H_2_ *via* the hy­droxy-O—H⋯N(pyrid­yl) hydrogen bonds was used as the input for calculations. A list of the short inter­atomic contacts discussed below is given in Table 2 ▸. Through this analysis, several red spots were identified on the *d*_norm_ surfaces, Fig. 3 ▸, of the individual ^4^ *L*H_2_ and BA mol­ecules, hereafter BA-I for the O3-containing mol­ecule and BA-II for the O5-mol­ecule, which indicate the presence of close contacts with distances shorter than the sum of the respective van der Waals radii (Spackman & Jayatilaka, 2009 ▸). Among all contacts, the terminal benzoic acid-O4—H4*O*⋯N1(pyrid­yl), benzoic acid-O6—H6*O*⋯N4(pyrid­yl), amide-N2—H2*N*⋯O2(amide) and amide-N3—H3*N*⋯O5(carbon­yl) hydrogen-bonding inter­actions exhibit the most intense red spots on the *d*_norm_ surfaces, suggestive of strong inter­actions.

Other, relatively less intense red spots in Fig. 3 ▸(*a*) and (*b*) [in the order of moderate intensity (*m*) to weak intensity (*w*)] were identified for C6—H6*B*⋯O1 (*m*), C12—H12⋯O3 (*m*), C2—H2⋯O5 (*m*) and C1—H1⋯O3 (*w*), Table 1 ▸, and C20—H20⋯C8 (*m*), C28—H28⋯O1 (*w*), C9—H9*B*⋯C17 (*w*), C9—H9*B*⋯O3 (*w*), C13—H13⋯C1 (*w*), C12⋯C21 (*w*) and C8⋯C26 (*w*), Table 2 ▸. With the exception of the moderately intense red spot observed for C20—H20⋯C8 as well as those with relatively weak intensity, the other contacts are consistent with the inter­actions detected through an analysis with *PLATON* (Spek, 2020 ▸). As for the two benzoic acid mol­ecules in the asymmetric unit, the contacts between them are established through C25—H25⋯C21, C27—H27⋯C18 as well as C24—H24⋯O4 inter­actions with diminutive intensity on the *d*_norm_ maps shown in Fig. 3 ▸(*c*) and (*d*).

**Figure 4:**
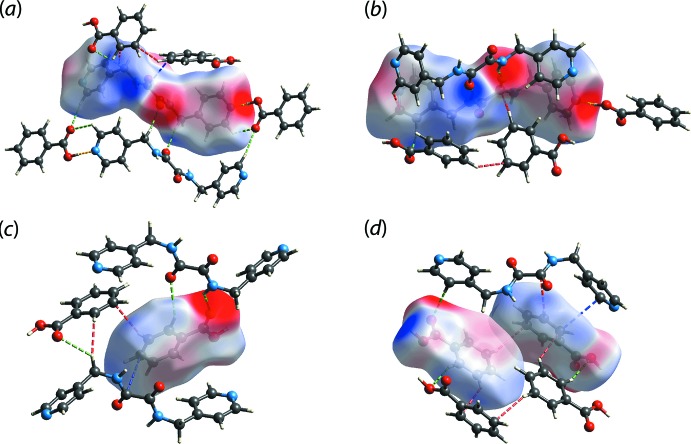
The electrostatic potential mapped onto the Hirshfeld surface within the isosurface range of −0.0562 to 0.0861 atomic units for (*a*) ^4^ *L*H_2_, (*b*) ^4^ *L*H_2_, (*c*) BA-II and (*d*) BA-II (left) and BA-I (right).

The electrostatic potential mapping was performed on the individual ^4^ *L*H_2_, BA-I and BA-II mol­ecules through DFT-B3LYP/6-31G(*d*,*p*) to further study the nature of the close contacts, Fig. 4 ▸. The results are consistent with the above in that the O*-*–H⋯N and N—H⋯O hydrogen-bonding contacts that exhibited the most intense red spots on the *d*_norm_ map are highly electrostatic in nature, as evidenced from the intense electronegative (red) and electropositive (blue) regions on the Hirshfeld surfaces of the individual mol­ecules. Other regions are relatively pale, indicating the complementary role of the remaining contacts in sustaining the mol­ecular network in the crystal.

**Figure 5:**
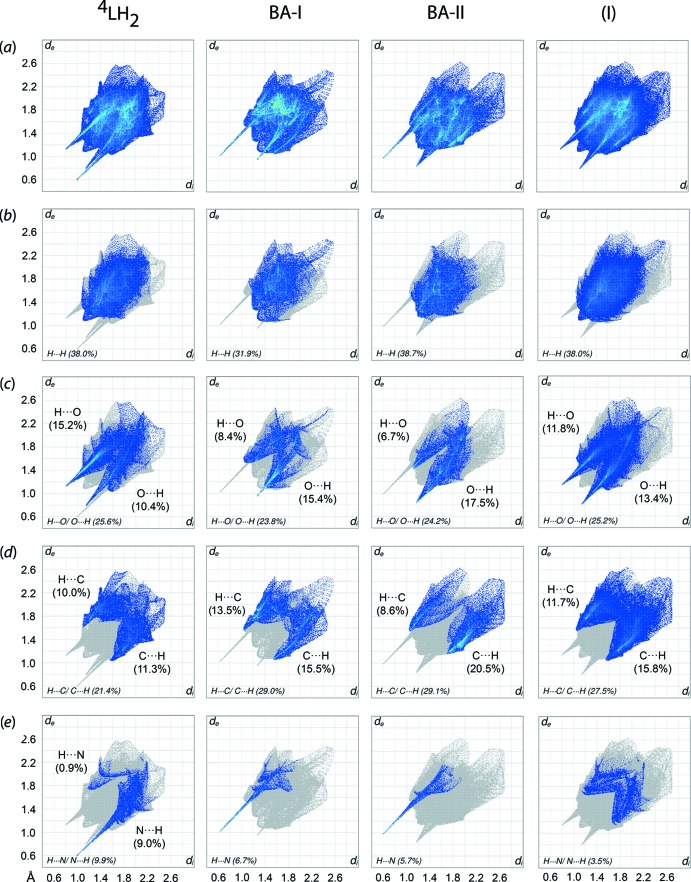
(*a*) The overall two-dimensional fingerprint plots for ^4^ *L*H_2_, BA-I, BA-II and the three-mol­ecule aggregate in (I), and those delineated into (*b*) H⋯H, (*c*) H⋯O/O⋯H, (*d*) H⋯C/C⋯H and (*e*) H⋯N/N⋯H contacts, with the percentage contributions specified in each plot.

The two-dimensional fingerprint plots were generated in order to qu­antify the close contacts for compound (I) overall, *i.e*. the three-mol­ecule aggregate specified above, as well as its individual ^4^ *L*H_2_, BA-I and BA-II components, Fig. 5 ▸. The overall fingerprint plot of (I) exhibits a shield-like profile with a pair of symmetric spikes and contrasts those for the indiv­idual components with asymmetric spikes, indicating the inter­dependency between ^4^ *L*H_2_ and benzoic acid in constructing the mol­ecular packing of the system, in contrast to the previously reported benzene monosolvate of ^4^ *L*H_2_ (Tan, Halcovitch *et al.*, 2019 ▸).

The major surface contacts for (I) can be split four ways: into H⋯H (38.0%), C⋯H/H⋯C (27.5%), O⋯H/H⋯O (25.2%) and N⋯H/H⋯N (3.5%) contacts. The distributions for H⋯H and N⋯H/H⋯N are evenly distributed between the inter­nal (*i.e.* the donor or acceptor atoms inter­nal to the surface) and external (*i.e.* the donor or acceptor atoms external to the surface) contacts. In contrast, for H⋯C/C⋯H and H⋯O/O⋯H, the distributions are slightly inclined towards (inter­nal)-C⋯H-(external) (15.8%) and (inter­nal)-O⋯H-(external) (13.4%) as compared to the corresponding counterparts at 11.7 and 11.8%, respectively. A detailed analysis of the *d*_i_ + *d*_e_ distances shows that the closest H⋯O/O⋯H and H⋯C/C⋯H contacts of ∼1.95 Å and ∼2.62 Å, respectively, occur at distances shorter than the sum of the respective van der Waals radii of 2.61 and 2.79 Å, while the H⋯H (∼2.20 Å) and N⋯H/ H⋯N (2.80 Å) contacts are longer than the sum of van der Waals radii of 2.18 and 2.64 Å, respectively.

The ^4^ *L*H_2_ mol­ecule also displays a shield-like profile with asymmetric spikes which upon further decomposition could be delineated into H⋯H (38.0%), H⋯O/O⋯H (25.6%), H⋯C/C⋯H (21.4%) and H⋯N/N⋯H (9.9%) contacts. The H⋯O/O⋯H contact exhibits a forceps-like profile with the distribution inclined towards inter­nal-H⋯O-external (15.2%) as compared to inter­nal-O⋯H-external (10.4%), and both with tips at *d*_i_ + *d*_e_ ∼1.94 Å which is indicative of significant hydrogen bonding. Similarly, the asymmetric, needle-like profile for the H⋯N/N⋯H contact is inclined towards the inter­nal-N⋯H-external (9.0%) with the tip at *d*_i_ + *d*_e_ = ∼1.6 Å, while the remaining 0.9% is attributed to the inter­nal-H⋯N-external contact with *d*_i_ + *d*_e_ of ∼2.94 Å (> sum of van der Waals radii). The H⋯C/C⋯H contacts are evenly distributed on both sides of the contacts with the *d*_i_ + *d*_e_ of ∼2.64 Å which is slightly shorter than the sum of van der Waals radii. On the other hand, the H⋯H contacts have little direct influence in sustaining the mol­ecular packing as shown from the shortest *d*_i_ + *d*_e_ value of ∼2.2 Å, which is longer than the sum of the van der Waals radii despite the prominent contributions these make to the overall surface

As for the pair of BA mol­ecules, both BA-I and BA-II possess similar, claw-like profiles which differ in the diffuse region, with the former being the characteristic of H⋯H contacts while the latter is due to H⋯C/C⋯H inter­actions. Qu­anti­tatively, differences mainly relate to the percentage contribution by H⋯H contacts, *i.e*. 31.9% for BA-I *cf*. 38.7% for BA-II. The discrepancy in the distribution for BA-I is compensated by the increase in O⋯C/C⋯O and C⋯C contacts with the distribution being 4.8 and 2.9%, respectively. The distribution for H⋯C/C⋯H (29.0 *vs* 29.1%), H⋯O/O⋯H (23.8 *vs* 24.2%) and H⋯N (6.7 *vs* 5.7%) contacts is approximately the same in both BA-I and BA-II, except that the H⋯C/C⋯H distribution for BA-II is significantly more inclined towards inter­nal-C⋯H-external (20.5%) than the inter­nal-H⋯C-external (8.6%) in contrast to the relatively balanced distribution for BA-I 15.5% for inter­nal-C⋯H-external *vs* 13.5% for inter­nal-H⋯C-external. In BA-I, the *d*_i_ + *d*_e_ values for H⋯O/O⋯H, H⋯C/C⋯H and H⋯N/N⋯H at the tips are ∼2.26–2.70, 2.62 and 1.64 Å, respectively, while the equivalent values for the analogous contacts for BA-II have tips at 2.02–2.56, 2.62–2.86 and 1.58 Å, respectively. Among these contact distances, the O⋯H, H⋯C/C⋯H and H⋯N for BA-I as well as H⋯O/O⋯H, H⋯C and H⋯N for BA-II are shorter than the sum of van der Waals radii. As expected, the minimum *d*_i_ + *d*_e_ value for the H⋯H contacts is longer than the sum of van der Waals radii, even if it is the most dominant contact for each mol­ecule. The aforementioned data for BA-I and BA-II clearly distinguishes the independent mol­ecules.

## Computational chemistry   

To assess the strength of the specified inter­actions in the Hirshfeld surface analysis, the mol­ecules in (I) were subjected to energy calculations through *CrystalExplorer17* (Turner *et al.*, 2017 ▸); the results are collated in Table 3 ▸. Among all close contacts present in (I), the pairwise inter­actions of the amide-N2—H2*N*⋯O2(amide) hydrogen bonds complemented by a pair of pyridyl-C13—H13⋯C1(pyrid­yl) inter­actions between two oxamide mol­ecules led to the greatest inter­action energy (*E*_tot_) of −75.2 kJ mol^−1^. This value is comparable to *E*_tot_ of −71.7 kJ mol^−1^ calculated for the classical eight-membered {⋯HOCO}_2_ inter­action (Tan & Tiekink, 2019*a* ▸). The second strongest inter­action arises from the hy­droxy-O4—H4*O*⋯N1(pyrid­yl) and pyridyl-C1—H1⋯O3(carbon­yl) contacts, which combine to generate a seven-membered heterosynthon with *E*_tot_ of −50.1 kJ mol^−1^. A diminution in *E*_tot_ is observed for the other pyridyl terminus, which only comprises a carb­oxy­lic-O6—H6*O*⋯N4(pyrid­yl) hydrogen bond without a supporting pyridyl-C—H⋯O(carbon­yl) inter­action, showing an energy of −43.9 kJ mol^−1^ and ranked third strongest among all inter­actions in (I).

The next highest inter­action energy with *E*_tot_ of −36.3 kJ mol^−1^ involves contributions from the amide-N3—H3*N*⋯O5(carb­oxy­lic acid) and phenyl-C28—H28⋯O1(amide) contacts. Other inter­actions include the methyl­ene-C6—H6*B*⋯O1(amide) contacts (–25.1 kJ mol^−1^), the combination of benzoic acid-C20—H20⋯C8(amide) and benzoic acid-C12⋯C21(pyrid­yl) (–21.0 kJ mol^−1^), amide-C8⋯C26(benzoic acid) (–19.4 kJ mol^−1^), pyridyl-C2—H2⋯O5(carb­oxy­lic acid) (–15.6 kJ mol^−1^), a combination of (pyridine meth­yl)-C9—H9*B*⋯O3(carb­oxy­lic acid) and methyl­ene-C9—H9*B*⋯C17(benzoic acid) (−14.7 kJ mol^−1^), as well as pyridyl-C12—H12⋯O3(carb­oxy­lic acid) (–8.3 kJ mol^−1^). Some inconsistencies are observed between the calculated *E*_tot_ and Hirshfeld surface analysis, particularly for C2—H2⋯O5 and C12—H12⋯O3. These inter­actions can be considered weak even though they possess a relatively short contact distance compared to the sum of van der Waals radii, as indicated from the moderately intense red spots on the Hirshfeld surface. The contradiction could arise as a result of the relatively high repulsion terms, which weaken the inter­action energy.

**Figure 6:**
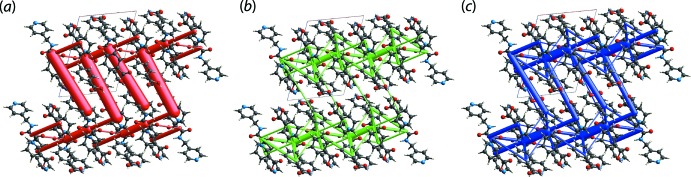
Perspective views of the energy frameworks of (I), showing the (*a*) electrostatic force, (*b*) dispersion force and (*c*) total energy. The cylindrical radius is proportional to the relative strength of the corresponding energies and they were adjusted to the same scale factor of 100 with a cut-off value of 8 kJ mol^−1^ within a 2 × 2 × 2 unit cells.

Overall, the crystal of (I) is mainly sustained by electrostatic forces owing to the strong ten-membered {⋯HNC_2_O}_2_ synthon as well as the terminal inter­actions between ^4^ *L*N_2_ and BA mol­ecules, through hy­droxy-O—H4⋯N(pyrid­yl) hydrogen bonds, lead to a zigzag electrostatic energy framework, Fig. 6 ▸(*a*). The packing system is further stabilized by the dispersion forces contributed by the ten-membered {⋯HNC_2_O}_2_ synthon complemented by other peripheral inter­actions such the pairwise C20—H20⋯C8/C12⋯C21, C8⋯C26 and C6—H6*B*⋯O1 inter­actions, which result in a dispersion energy framework resembling a spider web, Fig. 6 ▸(*b*). The combination of the electrostatic and dispersion forces leads to an overall energy framework that resembles a ladder, Fig. 6 ▸(*c*).

## Database survey   

As indicated in the *Chemical context*, ^4^ *L*H_2_ mol­ecules have long been known to form co-crystals with carb­oxy­lic acids. A list of ^4^ *L*H_2_/carb­oxy­lic acid co-crystals is given in Table 4 ▸, highlighting the symmetry of ^4^ *L*H_2_, the length of the central C—C bond, recognized as being long (Tiekink, 2017 ▸; Tan & Tiekink, 2020 ▸), and the O—H⋯N and NC—H⋯O (involving the C—H atom adjacent to the pyridyl-nitro­gen atom) separations associated with the hy­droxy-O—H⋯N(pyrid­yl) hydrogen bond. The data are separated into 1:1 and 1:2 ^4^ *L*H_2_:carb­oxy­lic acid species. In all cases, ^4^ *L*H_2_ adopts an anti-periplanar disposition of the pyridyl rings whereby the pyridyl rings lie to either side of the central C_2_N_2_O_2_ chromophore; often this is crystallographically imposed. This matches the situation in the two known polymorphs of ^4^ *L*H_2_ (Lee & Wang, 2007 ▸; Lee, 2010 ▸), but contrasts with the conformational diversity found in the isomeric ^3^ *L*H_2_ mol­ecules, *i.e*. in the polymorphs (Jotani *et al.*, 2016 ▸) and multi-component crystals (Tan & Tiekink, 2020 ▸). All but one structure forms hydroxyl-O—H⋯N(pyrid­yl) hydrogen bonds, involving both pyridyl rings, in their crystals. One reason put forward for the stability of hy­droxy-O—H⋯N(pyrid­yl) hydrogen bonds is the close approach of the pyridyl-C—H and carbonyl-O atoms to form a seven-membered {⋯O=COH⋯NCH} pseudo-synthon. In most, but not all examples, the carb­oxy­lic acid and pyridyl ring approach co-planarity, enabling the formation of the aforementioned pseudo-synthon. Among the co-crystals, only one example does not form the anti­cipated hydroxyl-O—H⋯N(pyrid­yl) hydrogen bonds. In this case, the co-former, *i.e*. 2-[(4-hy­droxy­phen­yl)diazen­yl]benzoic acid, carries a hydroxyl residue and this preferentially forms the hydrogen bonds to the pyridyl-N atoms. This observation is contrary to literature expectation where the carb­oxy­lic acid would be expected to form hydrogen bonds preferentially to pyridyl-N atom in instances where there is a competition with putative hydroxyl-O—H⋯N(pyrid­yl) hydrogen bonds (Shattock *et al.*, 2008 ▸). In this structure, the carb­oxy­lic acid is able to form an intra­molecular hy­droxy-O—H⋯N(azo) hydrogen bond to close an *S*(6) loop, in accord with Etter’s rules, *i.e*. ‘*six-membered ring intra­molecular hydrogen bonds form in preference to inter­molecular hydrogen bonds*’ (Etter, 1990 ▸). Finally, and for completeness, details for a salt are included in Table 4 ▸. Here, proton transfer has occurred, leading to a pyridinium-N—H⋯O(carboxyl­ate) hydrogen bond.

## Synthesis and crystallization   

The precursor, *N*,*N*′-bis­(pyridin-4-ylmeth­yl)oxalamide (^4^ *L*H_2_), was prepared according to the literature; M.p.: 486.3–487.6 K; lit. 486–487 K (Nguyen *et al.*, 1998 ▸). Reagent-grade benzoic acid (Merck) was used as received without further purification. Solid ^4^ *L*H_2_ (0.271 g, 0.001 mol) was mixed with benzoic acid (0.122 g, 0.001 mol) and the physical mixture was then ground for 15 min in the presence of a few drops of methanol. The procedures were repeated three times. Colourless blocks were obtained through careful layering of toluene (1 ml) on an *N*,*N*-di­methyl­formamide (1 ml) solution of the ground mixture. M.p.: 435.4–436 K. IR (cm^−1^): 3321 ν(N—H), 3070–2999 ν(C—H), 1702–1662 ν(C=O), 1506 ν(C=C), 1417 ν(C—N).

Similar experiments with ^4^ *L*H_2_:benzoic acid in molar ratios of 1:2, 1:3 and 1:4 were also attempted but only the 2:1 co-crystal (I) was isolated after recrystallization of the powders.

## Refinement   

Crystal data, data collection and structure refinement details are summarized in Table 5 ▸. The carbon-bound H atoms were placed in calculated positions (C—H = 0.95–0.99 Å) and were included in the refinement in the riding-model approximation, with *U*_iso_(H) set to 1.2*U*_eq_(C). The oxygen- and nitro­gen-bound H atoms were located from a difference Fourier map and refined with O—H = 0.84±0.01 Å and N—H = 0.88±0.01 Å, respectively, and with *U*_iso_(H) set to 1.5*U*_eq_(O) or 1.2*U*_eq_(N).

## Supplementary Material

Crystal structure: contains datablock(s) I, global. DOI: 10.1107/S2056989019016840/hb7875sup1.cif

Structure factors: contains datablock(s) I. DOI: 10.1107/S2056989019016840/hb7875Isup2.hkl

Supporting information file. DOI: 10.1107/S2056989019016840/hb7875Isup3.cml

CCDC reference: 1972449

Additional supporting information:  crystallographic information; 3D view; checkCIF report

## Figures and Tables

**Table 1:** Hydrogen-bond geometry (Å, °)

*D*—H⋯*A*	*D*—H	H⋯*A*	*D*⋯*A*	*D*—H⋯*A*
N2—H2*N*⋯O2	0.88 (1)	2.36 (1)	2.7192 (12)	105 (1)
N3—H3*N*⋯O1	0.88 (1)	2.36 (1)	2.7154 (12)	104 (1)
O4—H4*O*⋯N1^i^	0.86 (2)	1.78 (2)	2.6366 (12)	177 (2)
O6—H6*O*⋯N4^ii^	0.86 (2)	1.72 (2)	2.5731 (13)	169 (2)
N2—H2*N*⋯O2^iii^	0.88 (1)	2.05 (1)	2.8618 (12)	152 (1)
N3—H3*N*⋯O5^iv^	0.88 (1)	2.12 (1)	2.8516 (12)	140 (1)
C1—H1⋯O3^v^	0.95	2.58	3.2009 (14)	124
C2—H2⋯O5	0.95	2.47	3.3602 (14)	155
C6—H6*B*⋯O1^iv^	0.99	2.41	3.3826 (14)	166
C12—H12⋯O3^vi^	0.95	2.36	3.3025 (15)	171

Symmetry codes: (i) 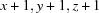; (ii) 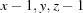; (iii) 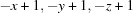; (iv) 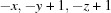; (v) 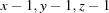; (vi) 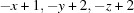.

**Table 2:** A summary of short inter­atomic contacts (Å) in (I)*^*a*^*

Contact	Distance	Symmetry operation
O2⋯H2*N^*b*^*	1.94	1 − *x*, 1 − *y*, 1 − *z*
C1⋯H13	2.79	−1 + *x*, *y*, −1 + *z*
N1⋯H4*O^*b*^*	1.65	−1 + *x*, −1 + *y*, −1 + *z*
O3⋯H1	2.50	1 + *x*, 1 + *y*, 1 + *z*
N4⋯H6*O^*b*^*	1.60	1 + *x*, *y*, 1 + *z*
O5⋯H3*N^*b*^*	2.02	−*x*, 1 − *y*, 1 − *z*
O1⋯H28	2.57	−*x*, 1 − *y*, 1 − *z*
O1⋯H6*B*	2.32	−*x*, 1 − *y*, 1 − *z*
C8⋯H20	2.61	*x*, *y*, *z*
C12⋯C21	3.39	*x*, *y*, *z*
C8⋯C26	3.35	*x*, −1 + *y*, *z*
O5⋯H2	2.35	*x*, *y*, *z*
O3⋯H9*B*	2.56	*x*, 1 + *y*, *z*
C17⋯H9*B*	2.69	*x*, 1 + *y*, *z*
O3⋯H12	2.23	1 − *x*, 2 − *y*, 2 − *z*
C21⋯H25	2.62	1 − *x*, 2 − *y*, 1 − *z*
C18⋯H27	2.67	*x*, *y*, *z*
O4⋯H24	2.58	1 − *x*, 2 − *y*, 1 − *z*

Notes: (*a*) The inter­atomic distances are calculated in *Crystal Explorer 17* (Turner *et al.*, 2017 ▸) whereby the *X*—H bond lengths are adjusted to their neutron values; (*b*) these inter­actions correspond to conventional hydrogen bonds.

**Table 3:** A summary of inter­action energies (kJ mol^−1^) calculated for (I)

Contact	*E*_ele_	*E*_pol_	*E*_dis_	*E*_rep_	*E*_tot_	Symmetry operation
N2—H2*N*⋯O2/						
C13—H13⋯C1	−60.9	−14.6	−58.5	82.5	−75.2	1 − *x*, 1 − *y*, 1 − *z*
O4—H4*O*⋯N1/						
C1—H1⋯O3	−90.7	−21.3	−13.0	118.0	−50.1	−1 + *x*, −1 + *y*, −1 + *z*
O6—H6*O*⋯N4	−95.2	−22.2	−11.3	134.4	−43.9	1 + *x*, *y*, 1 + *z*
N3—H3*N*⋯O5/						
C28—H28⋯O1	−32.8	−8.6	−16.1	30.3	−36.3	−*x*, 1 − *y*, 1 − *z*
C6—H6*B*⋯O1	−11.4	−5.1	−29.5	26.4	−25.1	−*x*, 1 − *y*, 1 − *z*
C20—H20⋯C8/						
C12⋯C21	−5.8	−1.5	−32.7	23.8	−21.0	*x*, *y*, *z*
C8⋯C26	−3.7	−1.0	−31.9	21.1	−19.4	*x*, −1 + *y*, *z*
C2—H2⋯O5	−9.4	−1.9	−14.0	12.9	−15.6	*x*, *y*, *z*
C9—H9*B*⋯O3/						
C9—H9*B*⋯C17	−6.0	−2.2	−17.3	13.5	−14.7	*x*, 1 + *y*, *z*
C12—H12⋯O3	−9.5	−2.4	−4.9	12.6	−8.3	1 − *x*, 2 − *y*, 2 − *z*

**Table 4:** Selected geometric data, *i.e.* central C—C bond length, O—H⋯N and NC—H⋯O(carbon­yl) separations (Å) for ^4^ *L*H_2_ in its co-crystals with carb­oxy­lic acids and salt with a carboxyl­ate anion

Carb­oxy­lic acid (CA)	Symmetry of ^4^ *L*H_2_	C—C	O—H⋯N(pyrid­yl)	NC—H⋯O(carbon­yl)	REFCODE	Reference
1:1 co-crystal						
bis­(carb­oxy­meth­yl)urea	–	1.53 (2)	1.73	2.54	CAJRAH	Nguyen *et al.* (2001 ▸)
			1.75	4.21		
diglycineoxamide	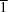	1.514 (5)	1.74	3.11	SEPSIP01	Nguyen *et al.* (2001 ▸)
poly(1,2-bis­(2-carb­oxy­eth­yl)tetra-1-en-3-yn-1,4-di­yl	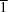	1.537 (13)	1.80	2.98	DOVSIR	Curtis *et al.* (2005 ▸)
						
2:1 co-crystal						
(4-nitro­phen­yl)acetic acid	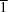	1.543 (2)	1.57	2.72	NAXMEG	Arman, Kaulgud *et al.* (2012 ▸)
benzoic acid	–	1.5401 (14)	1.67	2.59	–	This work
			1.72	3.46		
2-methyl­benzoic acid	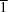	1.5356 (19)	1.79	2.60	WADXUX	Syed *et al.* (2016 ▸)
acetic acid^*a*^	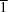	1.5397 (17)	1.75	2.81	GOQQIP	Tan & Tiekink (2019*b* ▸)
2-[(4-hy­droxy­phen­yl)diazen­yl]benzoic acid^*b*^	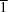	1.542 (2)	1.89	–	AJEZEV	Arman *et al.* (2009 ▸)
2,6-di­nitro­benzoate^*c*^	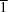	1.543 (3)	1.96^*c*^	2.51	TIPGUW	Arman, Miller *et al.* (2012 ▸)

Notes: (*a*) Characterized as a di-hydrate; (*b*) hy­droxy-O—N(pyrid­yl) hydrogen bond; (*c*) salt with a pyridinium-N—H⋯O(carboxyl­ate) hydrogen bond.

**Table 5:** Experimental details

Crystal data	
Chemical formula	C_14_H_14_N_4_O_2_·2C_7_H_6_O_2_
*M*_r_	514.53
Crystal system, space group	Triclinic, *P* 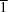
Temperature (K)	100
*a*, *b*, *c* (Å)	9.6543 (2), 9.9235 (2), 14.1670 (3)
α, β, γ (°)	100.755 (2), 108.318 (2), 95.617 (2)
*V* (Å^3^)	1247.90 (5)
*Z*	2
Radiation type	Cu *K*α
μ (mm^−1^)	0.81
Crystal size (mm)	0.12 × 0.07 × 0.05

Data collection	
Diffractometer	Rigaku XtaLAB Synergy Dualflex AtlasS2
Absorption correction	Gaussian (*CrysAlis PRO*; Rigaku OD, 2018 ▸)
*T*_min_, *T*_max_	0.832, 1.000
No. of measured, independent and observed [*I* > 2σ(*I*)] reflections	31534, 5220, 4736
*R*_int_	0.031
(sin θ/λ)_max_ (Å^−1^)	0.630

Refinement	
*R*[*F* ^2^ > 2σ(*F* ^2^)], *wR*(*F* ^2^), *S*	0.034, 0.093, 1.02
No. of reflections	5220
No. of parameters	359
No. of restraints	4
H-atom treatment	H atoms treated by a mixture of independent and constrained refinement
Δρ_max_, Δρ_min_ (e Å^−3^)	0.21, −0.28

Computer programs: *CrysAlis PRO* (Rigaku OD, 2018 ▸), *SHELXS* (Sheldrick, 2015*a* ▸), *SHELXL2017/1* (Sheldrick, 2015*b* ▸), *ORTEP-3 for Windows* (Farrugia, 2012 ▸), *DIAMOND* (Brandenburg, 2006 ▸) and *publCIF* (Westrip, 2010 ▸).

## supplementary crystallographic information

### Crystal data

**Table:**

C_14_H_14_N_4_O_2_·2C_7_H_6_O_2_	*Z* = 2
*M**_r_* = 514.53	*F*(000) = 540
Triclinic, *P*1	*D*_x_ = 1.369 Mg m^−^^3^
*a* = 9.6543 (2) Å	Cu *K*α radiation, λ = 1.54184 Å
*b* = 9.9235 (2) Å	Cell parameters from 16947 reflections
*c* = 14.1670 (3) Å	θ = 3.3–76.2°
α = 100.755 (2)°	µ = 0.81 mm^−^^1^
β = 108.318 (2)°	*T* = 100 K
γ = 95.617 (2)°	Prism, colourless
*V* = 1247.90 (5) Å^3^	0.12 × 0.07 × 0.05 mm

### Data collection

**Table:**

Rigaku XtaLAB Synergy Dualflex AtlasS2 diffractometer	5220 independent reflections
Radiation source: micro-focus sealed X-ray tube	4736 reflections with *I* > 2σ(*I*)
Detector resolution: 5.2558 pixels mm^-1^	*R*_int_ = 0.031
ω scans	θ_max_ = 76.4°, θ_min_ = 3.4°
Absorption correction: gaussian (CrysAlis PRO; Rigaku OD, 2018)	*h* = −12→12
*T*_min_ = 0.832, *T*_max_ = 1.000	*k* = −12→12
31534 measured reflections	*l* = −17→17

### Refinement

**Table:**

Refinement on *F*^2^	Primary atom site location: structure-invariant direct methods
Least-squares matrix: full	Secondary atom site location: difference Fourier map
*R*[*F*^2^ > 2σ(*F*^2^)] = 0.034	Hydrogen site location: mixed
*wR*(*F*^2^) = 0.093	H atoms treated by a mixture of independent and constrained refinement
*S* = 1.02	*w* = 1/[σ^2^(*F*_o_^2^) + (0.0539*P*)^2^ + 0.3396*P*] where *P* = (*F*_o_^2^ + 2*F*_c_^2^)/3
5220 reflections	(Δ/σ)_max_ = 0.001
359 parameters	Δρ_max_ = 0.21 e Å^−^^3^
4 restraints	Δρ_min_ = −0.28 e Å^−^^3^

### Special details

**Table:**

Geometry. All esds (except the esd in the dihedral angle between two l.s. planes) are estimated using the full covariance matrix. The cell esds are taken into account individually in the estimation of esds in distances, angles and torsion angles; correlations between esds in cell parameters are only used when they are defined by crystal symmetry. An approximate (isotropic) treatment of cell esds is used for estimating esds involving l.s. planes.

### Fractional atomic coordinates and isotropic or equivalent isotropic displacement parameters (Å^2^)

**Table:**

	*x*	*y*	*z*	*U*_iso_*/*U*_eq_	
O1	0.11169 (8)	0.35585 (8)	0.52445 (6)	0.02229 (17)	
O2	0.49733 (8)	0.39415 (8)	0.58292 (6)	0.02091 (16)	
N1	−0.09947 (10)	0.21060 (9)	0.09622 (7)	0.02157 (19)	
N2	0.25702 (9)	0.46333 (9)	0.45149 (6)	0.01791 (18)	
H2N	0.3484 (10)	0.4904 (14)	0.4548 (10)	0.024 (3)*	
N3	0.35581 (9)	0.31253 (9)	0.66765 (6)	0.01757 (18)	
H3N	0.2664 (11)	0.2971 (14)	0.6716 (11)	0.025 (3)*	
N4	0.75967 (10)	0.62970 (10)	0.97336 (7)	0.0225 (2)	
C1	−0.14959 (11)	0.32758 (11)	0.12456 (8)	0.0211 (2)	
H1	−0.240536	0.343746	0.080891	0.025*	
C2	−0.07474 (11)	0.42577 (11)	0.21457 (8)	0.0198 (2)	
H2	−0.113267	0.507935	0.231404	0.024*	
C3	0.05805 (11)	0.40266 (11)	0.28035 (8)	0.0178 (2)	
C4	0.11019 (11)	0.28206 (11)	0.25088 (8)	0.0209 (2)	
H4	0.200412	0.262855	0.293315	0.025*	
C5	0.02905 (12)	0.18954 (11)	0.15858 (8)	0.0226 (2)	
H5	0.066421	0.107775	0.138881	0.027*	
C6	0.13776 (11)	0.50952 (11)	0.37960 (8)	0.0190 (2)	
H6A	0.178912	0.594422	0.363694	0.023*	
H6B	0.064816	0.535030	0.412815	0.023*	
C7	0.23279 (11)	0.39410 (10)	0.51848 (7)	0.0168 (2)	
C8	0.37639 (11)	0.36670 (10)	0.59305 (7)	0.0165 (2)	
C9	0.47933 (11)	0.27763 (11)	0.74404 (8)	0.0198 (2)	
H9A	0.539689	0.227162	0.709171	0.024*	
H9B	0.439995	0.214321	0.779400	0.024*	
C10	0.57768 (11)	0.40374 (10)	0.82273 (7)	0.0179 (2)	
C11	0.51872 (12)	0.50271 (12)	0.87410 (9)	0.0261 (2)	
H11	0.414595	0.494664	0.858090	0.031*	
C12	0.61289 (13)	0.61374 (12)	0.94915 (9)	0.0274 (2)	
H12	0.571438	0.680499	0.984407	0.033*	
C13	0.81637 (12)	0.53568 (12)	0.92284 (8)	0.0242 (2)	
H13	0.920690	0.547791	0.938956	0.029*	
C14	0.73011 (12)	0.42117 (12)	0.84793 (8)	0.0224 (2)	
H14	0.774771	0.355655	0.814404	0.027*	
O3	0.55315 (9)	1.14822 (8)	0.95498 (6)	0.02525 (18)	
O4	0.72142 (9)	1.01211 (8)	0.94323 (6)	0.02496 (18)	
H4O	0.778 (2)	1.0790 (17)	0.9921 (12)	0.073 (6)*	
C15	0.58610 (11)	1.04164 (11)	0.91631 (8)	0.0187 (2)	
C16	0.47533 (12)	0.93433 (11)	0.82989 (8)	0.0190 (2)	
C17	0.32865 (12)	0.95458 (11)	0.79630 (8)	0.0212 (2)	
H17	0.299289	1.032279	0.830770	0.025*	
C18	0.22525 (12)	0.86117 (12)	0.71238 (9)	0.0251 (2)	
H18	0.125298	0.875244	0.689406	0.030*	
C19	0.26769 (13)	0.74743 (12)	0.66210 (9)	0.0277 (2)	
H19	0.197060	0.684161	0.604343	0.033*	
C20	0.41328 (14)	0.72607 (12)	0.69617 (9)	0.0285 (3)	
H20	0.442188	0.648021	0.661755	0.034*	
C21	0.51726 (12)	0.81880 (11)	0.78076 (9)	0.0234 (2)	
H21	0.616527	0.803107	0.804782	0.028*	
O5	−0.16140 (8)	0.74669 (8)	0.22439 (6)	0.02307 (17)	
O6	−0.03885 (9)	0.80244 (9)	0.12317 (6)	0.02892 (19)	
H6O	−0.1140 (19)	0.747 (2)	0.0769 (14)	0.088 (8)*	
C22	−0.05202 (11)	0.80401 (10)	0.21249 (8)	0.0187 (2)	
C23	0.08033 (11)	0.88364 (10)	0.30063 (8)	0.0180 (2)	
C24	0.20594 (11)	0.94266 (11)	0.28499 (8)	0.0192 (2)	
H24	0.209182	0.931865	0.217699	0.023*	
C25	0.32654 (12)	1.01739 (11)	0.36799 (8)	0.0220 (2)	
H25	0.412647	1.056907	0.357507	0.026*	
C26	0.32108 (12)	1.03425 (11)	0.46627 (8)	0.0238 (2)	
H26	0.402958	1.086590	0.522807	0.029*	
C27	0.19693 (13)	0.97515 (12)	0.48208 (8)	0.0263 (2)	
H27	0.193811	0.986854	0.549439	0.032*	
C28	0.07667 (12)	0.89864 (12)	0.39969 (8)	0.0234 (2)	
H28	−0.007838	0.856648	0.410794	0.028*	

### Atomic displacement parameters (Å^2^)

**Table:**

	*U*^11^	*U*^22^	*U*^33^	*U*^12^	*U*^13^	*U*^23^
O1	0.0163 (3)	0.0268 (4)	0.0250 (4)	0.0018 (3)	0.0078 (3)	0.0085 (3)
O2	0.0161 (3)	0.0251 (4)	0.0224 (4)	0.0018 (3)	0.0071 (3)	0.0073 (3)
N1	0.0217 (4)	0.0236 (4)	0.0177 (4)	0.0029 (3)	0.0053 (3)	0.0035 (3)
N2	0.0144 (4)	0.0222 (4)	0.0158 (4)	0.0011 (3)	0.0041 (3)	0.0041 (3)
N3	0.0153 (4)	0.0199 (4)	0.0159 (4)	0.0001 (3)	0.0042 (3)	0.0037 (3)
N4	0.0231 (4)	0.0248 (5)	0.0158 (4)	−0.0006 (4)	0.0035 (3)	0.0036 (3)
C1	0.0189 (5)	0.0246 (5)	0.0190 (5)	0.0039 (4)	0.0040 (4)	0.0072 (4)
C2	0.0193 (5)	0.0200 (5)	0.0205 (5)	0.0045 (4)	0.0064 (4)	0.0055 (4)
C3	0.0171 (5)	0.0206 (5)	0.0168 (5)	0.0014 (4)	0.0068 (4)	0.0058 (4)
C4	0.0189 (5)	0.0245 (5)	0.0188 (5)	0.0056 (4)	0.0050 (4)	0.0058 (4)
C5	0.0241 (5)	0.0230 (5)	0.0207 (5)	0.0072 (4)	0.0072 (4)	0.0039 (4)
C6	0.0187 (5)	0.0203 (5)	0.0171 (5)	0.0039 (4)	0.0045 (4)	0.0046 (4)
C7	0.0166 (5)	0.0166 (4)	0.0151 (4)	0.0012 (4)	0.0048 (4)	0.0008 (4)
C8	0.0166 (4)	0.0147 (4)	0.0154 (4)	0.0005 (3)	0.0041 (4)	0.0005 (4)
C9	0.0199 (5)	0.0194 (5)	0.0178 (5)	0.0021 (4)	0.0033 (4)	0.0051 (4)
C10	0.0193 (5)	0.0200 (5)	0.0138 (4)	0.0020 (4)	0.0038 (4)	0.0061 (4)
C11	0.0174 (5)	0.0309 (6)	0.0248 (5)	0.0033 (4)	0.0044 (4)	−0.0005 (5)
C12	0.0249 (5)	0.0285 (6)	0.0241 (5)	0.0046 (4)	0.0065 (4)	−0.0020 (4)
C13	0.0171 (5)	0.0319 (6)	0.0211 (5)	0.0005 (4)	0.0041 (4)	0.0058 (4)
C14	0.0213 (5)	0.0258 (5)	0.0199 (5)	0.0044 (4)	0.0077 (4)	0.0035 (4)
O3	0.0264 (4)	0.0233 (4)	0.0216 (4)	0.0062 (3)	0.0047 (3)	−0.0006 (3)
O4	0.0220 (4)	0.0262 (4)	0.0213 (4)	0.0050 (3)	0.0038 (3)	−0.0017 (3)
C15	0.0226 (5)	0.0211 (5)	0.0139 (4)	0.0036 (4)	0.0071 (4)	0.0057 (4)
C16	0.0239 (5)	0.0191 (5)	0.0151 (5)	0.0011 (4)	0.0077 (4)	0.0062 (4)
C17	0.0250 (5)	0.0207 (5)	0.0196 (5)	0.0028 (4)	0.0091 (4)	0.0067 (4)
C18	0.0233 (5)	0.0277 (6)	0.0231 (5)	−0.0008 (4)	0.0054 (4)	0.0095 (4)
C19	0.0312 (6)	0.0246 (5)	0.0219 (5)	−0.0066 (4)	0.0061 (4)	0.0030 (4)
C20	0.0346 (6)	0.0211 (5)	0.0271 (6)	−0.0005 (5)	0.0121 (5)	−0.0009 (4)
C21	0.0249 (5)	0.0216 (5)	0.0238 (5)	0.0025 (4)	0.0096 (4)	0.0042 (4)
O5	0.0201 (4)	0.0254 (4)	0.0238 (4)	0.0009 (3)	0.0082 (3)	0.0061 (3)
O6	0.0266 (4)	0.0374 (5)	0.0158 (4)	−0.0093 (3)	0.0058 (3)	−0.0004 (3)
C22	0.0203 (5)	0.0175 (5)	0.0187 (5)	0.0035 (4)	0.0070 (4)	0.0045 (4)
C23	0.0203 (5)	0.0158 (4)	0.0174 (5)	0.0041 (4)	0.0055 (4)	0.0038 (4)
C24	0.0216 (5)	0.0196 (5)	0.0171 (5)	0.0048 (4)	0.0067 (4)	0.0049 (4)
C25	0.0192 (5)	0.0218 (5)	0.0233 (5)	0.0023 (4)	0.0053 (4)	0.0050 (4)
C26	0.0234 (5)	0.0226 (5)	0.0194 (5)	0.0054 (4)	0.0010 (4)	0.0011 (4)
C27	0.0315 (6)	0.0310 (6)	0.0156 (5)	0.0068 (5)	0.0072 (4)	0.0042 (4)
C28	0.0251 (5)	0.0267 (5)	0.0205 (5)	0.0034 (4)	0.0102 (4)	0.0066 (4)

### Geometric parameters (Å, º)

**Table:**

O1—C7	1.2270 (12)	C13—H13	0.9500
O2—C8	1.2312 (12)	C14—H14	0.9500
N1—C5	1.3395 (14)	O3—C15	1.2162 (13)
N1—C1	1.3421 (14)	O4—C15	1.3197 (13)
N2—C7	1.3336 (13)	O4—H4O	0.860 (10)
N2—C6	1.4538 (13)	C15—C16	1.4980 (14)
N2—H2N	0.881 (9)	C16—C21	1.3902 (15)
N3—C8	1.3298 (13)	C16—C17	1.3929 (15)
N3—C9	1.4570 (13)	C17—C18	1.3895 (15)
N3—H3N	0.883 (9)	C17—H17	0.9500
N4—C12	1.3346 (15)	C18—C19	1.3867 (17)
N4—C13	1.3338 (15)	C18—H18	0.9500
C1—C2	1.3845 (15)	C19—C20	1.3869 (18)
C1—H1	0.9500	C19—H19	0.9500
C2—C3	1.3968 (14)	C20—C21	1.3935 (16)
C2—H2	0.9500	C20—H20	0.9500
C3—C4	1.3869 (15)	C21—H21	0.9500
C3—C6	1.5157 (14)	O5—C22	1.2237 (13)
C4—C5	1.3906 (15)	O6—C22	1.3084 (13)
C4—H4	0.9500	O6—H6O	0.865 (10)
C5—H5	0.9500	C22—C23	1.4991 (14)
C6—H6A	0.9900	C23—C24	1.3932 (15)
C6—H6B	0.9900	C23—C28	1.3955 (15)
C7—C8	1.5402 (14)	C24—C25	1.3904 (15)
C9—C10	1.5139 (14)	C24—H24	0.9500
C9—H9A	0.9900	C25—C26	1.3893 (16)
C9—H9B	0.9900	C25—H25	0.9500
C10—C11	1.3861 (15)	C26—C27	1.3824 (17)
C10—C14	1.3857 (15)	C26—H26	0.9500
C11—C12	1.3894 (16)	C27—C28	1.3899 (16)
C11—H11	0.9500	C27—H27	0.9500
C12—H12	0.9500	C28—H28	0.9500
C13—C14	1.3857 (15)		
			
C5—N1—C1	117.75 (9)	N4—C13—C14	123.03 (10)
C7—N2—C6	121.82 (9)	N4—C13—H13	118.5
C7—N2—H2N	119.7 (9)	C14—C13—H13	118.5
C6—N2—H2N	118.1 (9)	C13—C14—C10	118.93 (10)
C8—N3—C9	120.98 (9)	C13—C14—H14	120.5
C8—N3—H3N	120.2 (9)	C10—C14—H14	120.5
C9—N3—H3N	118.8 (9)	C15—O4—H4O	108.5 (15)
C12—N4—C13	118.30 (9)	O3—C15—O4	123.81 (9)
N1—C1—C2	122.96 (10)	O3—C15—C16	122.06 (10)
N1—C1—H1	118.5	O4—C15—C16	114.08 (9)
C2—C1—H1	118.5	C21—C16—C17	119.87 (10)
C1—C2—C3	119.25 (10)	C21—C16—C15	121.37 (10)
C1—C2—H2	120.4	C17—C16—C15	118.72 (9)
C3—C2—H2	120.4	C16—C17—C18	120.01 (10)
C4—C3—C2	117.79 (9)	C16—C17—H17	120.0
C4—C3—C6	123.35 (9)	C18—C17—H17	120.0
C2—C3—C6	118.85 (9)	C19—C18—C17	120.12 (11)
C3—C4—C5	119.32 (10)	C19—C18—H18	119.9
C3—C4—H4	120.3	C17—C18—H18	119.9
C5—C4—H4	120.3	C18—C19—C20	119.98 (10)
N1—C5—C4	122.91 (10)	C18—C19—H19	120.0
N1—C5—H5	118.5	C20—C19—H19	120.0
C4—C5—H5	118.5	C19—C20—C21	120.18 (11)
N2—C6—C3	114.41 (8)	C19—C20—H20	119.9
N2—C6—H6A	108.7	C21—C20—H20	119.9
C3—C6—H6A	108.7	C16—C21—C20	119.82 (11)
N2—C6—H6B	108.7	C16—C21—H21	120.1
C3—C6—H6B	108.7	C20—C21—H21	120.1
H6A—C6—H6B	107.6	C22—O6—H6O	108.4 (17)
O1—C7—N2	125.93 (9)	O5—C22—O6	123.94 (9)
O1—C7—C8	121.14 (9)	O5—C22—C23	122.33 (9)
N2—C7—C8	112.92 (8)	O6—C22—C23	113.73 (9)
O2—C8—N3	124.54 (9)	C24—C23—C28	119.83 (9)
O2—C8—C7	121.86 (9)	C24—C23—C22	121.07 (9)
N3—C8—C7	113.60 (9)	C28—C23—C22	119.10 (9)
N3—C9—C10	113.22 (8)	C25—C24—C23	119.91 (10)
N3—C9—H9A	108.9	C25—C24—H24	120.0
C10—C9—H9A	108.9	C23—C24—H24	120.0
N3—C9—H9B	108.9	C26—C25—C24	119.98 (10)
C10—C9—H9B	108.9	C26—C25—H25	120.0
H9A—C9—H9B	107.7	C24—C25—H25	120.0
C11—C10—C14	117.99 (9)	C27—C26—C25	120.23 (10)
C11—C10—C9	121.18 (9)	C27—C26—H26	119.9
C14—C10—C9	120.79 (9)	C25—C26—H26	119.9
C10—C11—C12	119.57 (10)	C26—C27—C28	120.18 (10)
C10—C11—H11	120.2	C26—C27—H27	119.9
C12—C11—H11	120.2	C28—C27—H27	119.9
N4—C12—C11	122.16 (11)	C27—C28—C23	119.85 (10)
N4—C12—H12	118.9	C27—C28—H28	120.1
C11—C12—H12	118.9	C23—C28—H28	120.1
			
C5—N1—C1—C2	−0.12 (16)	N4—C13—C14—C10	1.01 (17)
N1—C1—C2—C3	−0.91 (16)	C11—C10—C14—C13	0.07 (16)
C1—C2—C3—C4	1.13 (15)	C9—C10—C14—C13	−177.84 (10)
C1—C2—C3—C6	−178.91 (9)	O3—C15—C16—C21	174.45 (10)
C2—C3—C4—C5	−0.39 (15)	O4—C15—C16—C21	−3.20 (14)
C6—C3—C4—C5	179.65 (10)	O3—C15—C16—C17	−3.13 (15)
C1—N1—C5—C4	0.91 (16)	O4—C15—C16—C17	179.22 (9)
C3—C4—C5—N1	−0.66 (17)	C21—C16—C17—C18	−1.48 (15)
C7—N2—C6—C3	−87.36 (12)	C15—C16—C17—C18	176.13 (9)
C4—C3—C6—N2	−12.00 (14)	C16—C17—C18—C19	0.20 (16)
C2—C3—C6—N2	168.05 (9)	C17—C18—C19—C20	0.61 (17)
C6—N2—C7—O1	3.86 (16)	C18—C19—C20—C21	−0.12 (18)
C6—N2—C7—C8	−175.03 (8)	C17—C16—C21—C20	1.96 (16)
C9—N3—C8—O2	−1.50 (15)	C15—C16—C21—C20	−175.59 (10)
C9—N3—C8—C7	178.88 (8)	C19—C20—C21—C16	−1.17 (17)
O1—C7—C8—O2	173.94 (9)	O5—C22—C23—C24	177.14 (10)
N2—C7—C8—O2	−7.10 (13)	O6—C22—C23—C24	−3.01 (14)
O1—C7—C8—N3	−6.43 (13)	O5—C22—C23—C28	−2.98 (16)
N2—C7—C8—N3	172.52 (8)	O6—C22—C23—C28	176.87 (10)
C8—N3—C9—C10	75.45 (12)	C28—C23—C24—C25	−0.63 (15)
N3—C9—C10—C11	50.59 (14)	C22—C23—C24—C25	179.25 (9)
N3—C9—C10—C14	−131.57 (10)	C23—C24—C25—C26	−0.63 (16)
C14—C10—C11—C12	−0.84 (17)	C24—C25—C26—C27	1.00 (16)
C9—C10—C11—C12	177.05 (10)	C25—C26—C27—C28	−0.10 (17)
C13—N4—C12—C11	0.40 (18)	C26—C27—C28—C23	−1.16 (17)
C10—C11—C12—N4	0.64 (19)	C24—C23—C28—C27	1.52 (16)
C12—N4—C13—C14	−1.24 (17)	C22—C23—C28—C27	−178.36 (10)

### Hydrogen-bond geometry (Å, º)

**Table:**

*D*—H···*A*	*D*—H	H···*A*	*D*···*A*	*D*—H···*A*
N2—H2*N*···O2	0.88 (1)	2.36 (1)	2.7192 (12)	105 (1)
N3—H3*N*···O1	0.88 (1)	2.36 (1)	2.7154 (12)	104 (1)
O4—H4*O*···N1^i^	0.86 (2)	1.78 (2)	2.6366 (12)	177 (2)
O6—H6*O*···N4^ii^	0.86 (2)	1.72 (2)	2.5731 (13)	169 (2)
N2—H2*N*···O2^iii^	0.88 (1)	2.05 (1)	2.8618 (12)	152 (1)
N3—H3*N*···O5^iv^	0.88 (1)	2.12 (1)	2.8516 (12)	140 (1)
C1—H1···O3^v^	0.95	2.58	3.2009 (14)	124
C2—H2···O5	0.95	2.47	3.3602 (14)	155
C6—H6*B*···O1^iv^	0.99	2.41	3.3826 (14)	166
C12—H12···O3^vi^	0.95	2.36	3.3025 (15)	171

Symmetry codes: (i) *x*+1, *y*+1, *z*+1; (ii) *x*−1, *y*, *z*−1; (iii) −*x*+1, −*y*+1, −*z*+1; (iv) −*x*, −*y*+1, −*z*+1; (v) *x*−1, *y*−1, *z*−1; (vi) −*x*+1, −*y*+2, −*z*+2.
